# Progress in Surgery

**Published:** 1898-10-01

**Authors:** 


### Progress in Surgery.

### ABDOMINAL SURGERY AND SURGERY OF THE PERITONEUM.

Brothers[1] says that in dealing with pus in the peritoneal cavity it is well to spend some time in the introduction of pads to push the intestines out of the way. Even if pus escapes it will then do little mischief, provided the escaping matter is caught in the gauze. He has been so well satisfied with this plan that he has entirely dispensed with intra-abdominal flushings, and only uses drainage in doubtful cases. As a rule, if hæmorrhage is entirely controlled and any septic material has been thoroughly removed, it will be quite safe to close the wound without drainage.

In discussing ruptures of the viscera and their relation to surgical shock Dwight[2] states that the lesions of various organs in all degrees of severity have one result, which they present to a greater or less degree in common, viz., hæmorrhage, and that death in all cases, except in those which—like the intestines, bladder, and heart—may result fatally in a way peculiar to themselves, comes as a result of that hæmorrhage. Shock is present to a greater or less extent in nearly every case after accidents, which not infrequently result in ruptures of the viscera, and if it is true that in most cases the group of symptoms commonly described by the word shock is due to hæmorrhage, it is of the greatest importance that such patients should be treated for concealed hæmorrhage and not for shock.

In the management of cases of laparotomy the following points are important, according to Wiggin[3]: (1) Whenever practicable a prolonged preparatory treatment of patients is desirable. (2) Cathartics should be administered in the early part of this period, followed by large enemas, for the purpose of cleansing the intestinal tract. (3) A record of the body temperature, respirations, and pulse rate should be kept for several days before the operation, and a final examination of the urine should be made. (4) In the case of females operation should be performed a few days after the menstrual period, and the vagina should be cleansed even when it is intended that the operation shall be by the abdominal route only. (5) The administration of a small quantity of peptonised food (one ounce) containing stimulants two hours before giving the anæsthetic, for the purpose of lessening the tendency to nausea and vomiting after the recovery of consciousness. (6) The anæsthetic should be given by an experienced physician in the smallest possible quantity. (7) The patient's body should be properly protected with clothing and blankets during the operation. (8) The patient should be stimulated before the heart has become much exhausted, and intravenous saline injections used before the radial pulse has become extinct. (9) After free irrigation, a quantity of hot saline solution should be left in the abdominal cavity for the purpose of stimulating the patient, preventing the formation of intestinal adhesions, and lessening the danger of septic infection of the peritoneum. (10) For the first two days of convalescence the patient must be made comfortable by change of position and by the use of the rectal tube. (11) Food should be administered early and in reasonable quantities. (12) Stimulating enemata should be withheld after operations in which extensive and firm pelvic adhesions have been broken up. (13) The question of reopening the peritoneal cavity in a case of supposed concealed hæmorrhage should be considered. (14) The stomach should be washed out as soon as the diagnosis of intestinal paresis is made, and the use of saline cathartics should be persisted in until the bowels move. (15) Cathartics should not be administered too early or in too large doses in those cases who are pursuing a normal convalescence.

**Draining the Peritoneal Cavity** by ordinary methods is considered by Delagénière[4] to be defective, and he proposes to drain the abdomen in a similar way to that of a spirit lamp by its wick. He employs a perforated nickel tube, in which is inserted a skein of cotton. This skein closely fits the interior of the metallic tube, and is frayed out as it projects from either end. Both the outer tube and the skein can be sterilised, and the skein can be changed without moving the tube; but in no case should the metal tube be allowed to remain for more than 36 hours. The nickel tubes used by the author—who has obtained excellent results—vary in length from 8 to 10 cm., and in diameter from 15 to 20 mm.

**Abdominal Section as a Cure for Vomiting** is borne out in some recent cases. Naylor[5] reports a case of vomiting for 18 years, which was cured by the separation of adhesions in the neighbourhood of the stomach after laparotomy. He operated because of a supposed pyloric stenosis, but, instead of this, he found adhesions tying the stomach down above and below, and these had probably produced spasmodic vomiting and tightening of the pylorus. All adhesions which could be, without danger, were broken up, and the stomach was freed in all directions except towards the liver, where the adhesions were too firm and deep.

**Peritoneal Tuberculosis** is discussed by Sims,[6] who enumerates some of the theories as to the production of cure in these cases by laparotomy as follows: (1) It has been claimed that the good result has been due to the use of chemical disinfectants; (2) the result has been attributed to the use of drainage; (3) some claim that cure has been produced by the exposure of the abdominal cavity to sunlight and air; (4) that cure depends upon the removal of ascitic fluid and the consequent altered blood circulation; (5) that by the accidental introduction of bacteria a toxalbumin is produced which is fatal to the tubercle bacilli; (6) that the opening of the abdomen and the operative handling of the organs constitute a traumatism, which establishes a fibrinous peritonitis, resulting in the encapsulation of the tubercle bacilli and their arrest; (7) that the cure is owing to the advent of leucocytes and is the result of phagocytosis; and (8) that the mere opening of the abdominal cavity by an incision brings about a physiological change in the peritoneum, which in some way makes it cease to be a proper soil for the growth of the tubercle bacilli. No one of these theories can as yet be demonstrated to be true, and some of them may readily be discarded. The author thinks we are forced to the conclusion that the opening of the abdomen produces a change in the physiological character of the peritoneum, which makes it overcome and destroy the tubercle bacillus; but this is merely begging the question, and he feels that the subject is still wanting in elucidation. His own conclusions on laparotomy for tuberculous disease of the peritoneum are as follows: (1) That the danger of the operation is very slight (the death-rate being below 3 per cent.) (2) That sepsis is not so likely to occur in these cases as in laparotomy on healthy peritonea, on account of the pathological changes which have taken place in the membrane. (3) That tuberculous infection of the wound does not occur. (4) That disinfectants are useless, and that drainage should not be used, as it is likely to result in a permanent sinus. (5) That in unsuccessful cases the operation at least does no harm. Most of the patients who have died after the operation have succumbed to a general tuberculosis, or to tuberculosis of some other organ. (6) That established—not advanced—pulmonary tuberculosis is an indication for and not against the operation; for the improvement gained enables the patient better to resist the phthisis, and if this latter be but incipient, recovery may take place. (7) That laparotomy is the proper form of treatment for these cases. Marked improvement occurs in about 80 per cent. of cases, and a permanent cure is effected in about 30 per cent. of all cases operated upon. In operating, the incision should be large enough to allow satisfactory exploration of the abdominal cavity. When possible the original focus of the disease should be removed. This will often be the case when the female pelvic organs or the appendix vermiformis is the site. He is convinced that the more simply and rapidly the entire operation is performed the better will be the result, and advises against irrigation or attempted medication of the abdominal cavity. When hydrops exist the fluid should be fully evacuated. When there is an encysted mass consisting of fluid or of granulation tissue, it is well to separate the adhesions and carefully sponge out the cavity, provided too much tearing and bleeding will not result; otherwise, it is better not to make such manipulation.

Watson Cheyne[7] records an interesting case of a tuberculous abscess in the abdomen communicating with the rectum. When the inflammatory tissue surrounding the abscess cavity had been peeled off from the uterus, Douglas's pouch and the rectum, it was found that there was a large hole in the rectum which almost admitted the little finger. Owing to the great depth of the parts, no attempt was made to stitch up the hole in the rectum. The pelvis having been sponged out, five large drainage tubes were inserted, these extending right down to the hole in the rectum, and the rest of the wound was then stitched up and an antiseptic dressing applied. The patient made a good recovery. The interest of the case is that it shows the possibility of removing a septic mass without infecting the peritoneal cavity, and more especially the possibility of leaving an opening of considerable size communicating freely with the rectum and the peritoneum without the occurrence of any subsequent peritonitis.

**Acute Septic Peritonitis** is treated of by Lockwood, who says, with regard to operation in these cases, that the routine procedure in a very acute localised septic peritonitis is to cut down and open the abscess at once—not to waste any time in looking for the appendix or tubes; but if there are not many adhesions a grangrenous appendix should be seized and removed. When there is a single abscess the adhesions should not be disturbed, so that fresh tracks in the peritoneum may not be opened up for the invasion of the pus. In these cases cleaning out the abscess cavity with swabs or sponges is probably sufficient; but where the abscess cavity is not so limited the author uses irrigation with extreme freedom. For this purpose he prefers strong solutions of biniodide of mercury.

**Retro-Peritoneal Lipoma** in a child aged eight years is recorded by Dalziel.[9] It weighed 13 lb., and caused an enormous projection of the abdomen. An attempt was made at removal, but the patient died suddenly of syncope before the incision was completed. The great majority of these cases have not been diagnosed before operation, and only 4 per cent. of retro-peritoneal lipomata are prevertebral in origin, as in this case.

[1] Med. Rec., April 30, 1898, p. 624. [2] Therap. Gaz, Jan., 1898, p. 27. [3] Lancet, April 30, 1898, p. 1,173. [4] Brit. Med. Jour., April 30, 1898, and Bull. et Mém. de la Soc. de Chir., No. 12, 1898. [5] Indian Med. Rec., Feb. 1, 1898, p. 101, and Amer. Med. Surg. Bull., Mar. 25, 1898, p. 275. [6] Med. Rec., April 2, 1898, p. 475. [7] Lancet, May 7, 1898, p. 1,255. [8] Clin. Jour., June 8, 1898, p. 129. [9] Glasgow Med. Jour., May, 1898 p. 372.

